# Obstetric fistula in southern Mozambique: a qualitative study on women’s experiences of care pregnancy, delivery and post-partum

**DOI:** 10.1186/s12978-020-0860-0

**Published:** 2020-01-31

**Authors:** Helena Boene, Sibone Mocumbi, Ulf Högberg, Claudia Hanson, Anifa Valá, Anna Bergström, Esperança Sevene, Khátia Munguambe

**Affiliations:** 1 Centro de Investigação em Saúde de Manhiça (CISM), Rua 12, Vila da Manhiça, 1121 Manhiça, Mozambique; 2 Department of Obstetrics and Gynaecology, Faculty of Medicine, Universidade Eduardo Mondlane (UEM), Av. Agostinho Neto 679, 1100 Maputo, Mozambique; 3 Department of Women’s and Children’s Health, Women’s and Children’s Health, Uppsala University, Akademiska sjukhuset, SE-75185 Uppsala, Sweden; 4 Department of Public Health Sciences, Karolinska Institutet, Tomtebodavagen 18A, Plan 4, Stockholm, Sweden; 5 Department of Disease Control, London School of Hygiene and Tropical Medicine, Keppel St, London, WC1E 7HT UK; 6 University College London, Institute for Global Health, Gower St, London, WC1E 6BT UK; 7 Department of Physiological Science, Clinical Pharmacology, Faculty of Medicine, UEM, Av. Salvador Allende 702 R/C, Maputo, Mozambique; 8 Department of Community Health, Faculty of Medicine, UEM, Av. Salvador Allende 702 R/C, Maputo, Mozambique

**Keywords:** Obstetric fistula, women’s experiences, Quality of care, Mozambique

## Abstract

**Background:**

Obstetric fistula is still common in low- and middle-income countries (LMIC) despite the on-going shift to increased facility deliveries in the same settings. The social behavioural circumstances in which fistula, as well as its consequences, still occur are poorly documented, particularly from the perspective of the experiences of women with obstetric fistula. This study sought to describe women’s experiences of antenatal, partum and post-partum care in southern Mozambique, and to pinpoint those experiences that are unique to women with fistula in order to understand the care-seeking and care provision circumstances which could have been modified to avoid or mitigate the onset or consequences of fistula.

**Methods:**

This study took place in Maputo and Gaza provinces, southern Mozambique, in 2016–2017. Qualitative data were collected through in-depth interviews conducted with 14 women with positive diagnoses of fistula and an equal number of women without fistula. All interviews were audio-recorded and transcribed verbatim prior to thematic analysis using NVivo11.

**Results:**

Study participants had all attended antenatal care (ANC) visits and had prepared for a facility birth. Prolonged or obstructed labour, multiple referrals, and delays in receiving secondary and tertiary health care were common among the discourses of women with fistula. The term “fistula” was rarely known among participants, but the condition (referred to as “loss of water” or “illness of spillage”) was recognised after being prompted on its signs and symptoms. Women with fistula were invariably aware of the links between fistula and poor birth assistance, in contrast with those without fistula, who blamed the condition on women’s physiological and behavioural characteristics.

**Conclusion:**

Although women do seek antenatal and peri-partum care in health facilities, deficiencies and delays in birth assistance, referral and life-saving interventions were commonly reported by women with fistula. Furthermore, weaknesses in quality of care, not only in relation to prevention, but also the resolution of the damage, were evident. Quality improvement of birth care is necessary, both at primary and referral level. There is a need to increase awareness and develop guidelines for prevention, early detection and management of obstetric fistula, including early postpartum treatment, availability of fistula repair for complex cases, and rehabilitation, coupled with the promotion of community consciousness of the problem.

## Resumo em Português

**Antecedentes:**

A fístula obstétrica continua a ser um problema de saúde comum em países de baixa e média renda, apesar das tendências de aumento dos partos institucionais nesses países. As circunstâncias socio-comportamentais em que a fístula ocorre, assim como suas consequências, são pouco documentadas, principalmente do ponto de vista das próprias mulheres com fístula obstétrica. Este estudo descreveu as experiências das mulheres em relação aos cuidados pré-natais, de parto e pós-parto no sul de Moçambique e identificou as experiências particulares das mulheres com fístula, a fim de compreender as circunstâncias em que a procura e oferta de cuidados poderiam ter sido modificadas para evitar ou mitigar a contracção ou as consequências da fístula.

**Métodos:**

Este estudo foi realizado nas províncias de Maputo e Gaza, sul de Moçambique, em 2016–2017. Os dados qualitativos foram colhidos através de entrevistas em profundidade realizadas com 14 mulheres com diagnóstico positivo de fístula e um número igual de mulheres sem fístula. Todas as entrevistas foram gravadas em áudio e transcritas na íntegra para posterior análise temática utilizando NVivo11.

**Conclusão:**

Embora as mulheres procurem cuidados pré-natais e peri-parto nas unidades sanitárias, deficiências e atrasos na assistência ao parto, referência e na indicação e aplicação de intervenções vitais foram experiências comumente relatadas por mulheres com fístula. Além disso, foram evidentes fragilidades na qualidade da assistência, não só em relação à prevenção, mas também à resolução dos danos. É crucial melhorar a qualidade da assistência ao parto, tanto ao nível primário como nos de referência. Existe a necessidade de aumentar a conscientização e desenvolver diretrizes para prevenção, detecção precoce e manejo da fístula obstétrica, incluindo tratamento pós-parto precoce, disponibilidade de reparo da fístula para casos complexos e reabilitação, juntamente com a promoção do conhecimento da comunidade sobre o problema.

## Plain English summary

Obstetric fistula is an abnormal opening between the vagina and the bladder or anus, that happens during prolonged labour or obstructed childbirth, and causes uncontrolled passing of urine or faeces. It is still common in poorly resourced countries and there is little information available about the situations when women experience fistula and its consequences. Therefore, it is important to further understand the knowledge and experiences of women with obstetric fistula. This study aimed to describe women’s childbirth experiences in southern Mozambique and their knowledge about fistula, and to highlight those that are unique to women who ended up with fistula in order to pinpoint the attitudes and practices that could have been changed to prevent fistula.

We interviewed 14 women with fistula and 14 women without fistula in two provinces in southern Mozambique. Although they sought antenatal care and gave birth in health facilities, women with fistula reported experiencing delayed referral during prolonged labour and slow decision-making regarding the provision of the best possible treatment. While women with fistula showed awareness of the causes of this problem, those without fistula made blameful and discriminatory statements about this condition. There is a need for an improvement in knowledge about fistula and its causes and consequences among community members. There is also the need to improve the promotion of actions meant for women at risk of birth complications so that they are treated in time and that healthcare staff have a better capacity to diagnose and care for women with fistula at the primary level.

## Background

Obstetric fistula is an abnormal opening between the vagina and bladder and/or rectum that causes uncontrollable and continuous leakage of urine and/or faeces with devastating consequences for the women who are affected by this condition [1]. Obstetric fistula is due to tissue ischemia and necrosis in the birth canal, which can also cause nerve damage, caused by continuous compression of the foetal presentation during prolonged and obstructed labour, but its cause may also be iatrogenic [2].

There is a clear link between obstetric fistula and delays in seeking, reaching or receiving adequate birth care (the three delays) [3–5]. The problem is exacerbated by deficient postpartum care, which includes delayed diagnosis and lack of prompt treatment through procedures as simple as a catheter to resolve a small fistula [6], andL appropriate care for complex cases [7].

Despite increases in facility delivery, severe adverse outcomes of pregnancy, including fistula, are common in LMIC [8–10]. A meta-analysis reports a prevalence of 1.57 per 1000 women in sub-Saharan Africa [11], and a recent population-based study in southern Mozambique, in a setting with 87% facility birth, showed that fistula incidence was 1.1 per 1000 in recently pregnant women [12]. Hence, in this changing landscape of few home births but a persisting high incidence of obstetric fistula [11], studies addressing women’s experiences of contracting and living with obstetric fistula are required to better understand the context in which such fistula are contracted and to raise awareness among health professionals, in particular, and communities, in general, regarding prevention, early detection, and care and treatment for those affected by this health problem.

The body of literature on women living with an obstetric fistula is growing. There is new knowledge deriving from qualitative studies targeting women with obstetric fistulas and focusing on the impact of fistula on women’s lives [13, 14]. Most of the literature states that the physiological sexual and reproductive repercussions of fistula lead to negative psychological, social and economic consequences, with an emphasis on stigma [3, 15]. Women with fistula are usually blamed for the constant smell of their excreta and suffer rejection and shunning from their husbands or partners, relatives, and the wider communities [16].

The majority of the existing studies seem to have intended to highlight the dramatic distress, mostly at social level, of living with fistula. Some of these studies listed the major issues that women with incontinence face, but few examined the women’s perspectives of the onset of the damage and their coping experiences, even prior to accessing adequate care, as the majority of them exclusively approached those living with the devastating consequences of fistulas, but who were already receiving care or in the process of undergoing surgical repair services at the time of recruitment [13, 14]. Few studies followed a population approach to allow the identification of untreated cases in the community.

The aim of this population-based study is twofold: (1) to describe the unique views and experiences of women living with obstetric fistula, including antenatal care, birth arrangements, sought and obtained birth assistance and post-natal care, their coping mechanisms from the onset of the obstetric fistula, as well as the physical, psychological and social consequences of this health problem; and (2) to explore the perceptions of fistula and attitudes towards women with fistula among women who are not living with fistula.

## Methods

### Study setting and participants

This study was nested in an epidemiologic study assessing the incidence of obstetric fistula in Maputo and Gaza provinces, both in southern Mozambique [12]. These two provinces, which accommodate 3,953,752 people altogether [17], are characterized as being typically rural, with extended pockets of impoverished areas, where agriculture, livestock rearing, informal trading, migrant labour (mainly to South Africa), handicrafts, and work in private sugar and rice processing farms are the principal occupations [18]. Primary healthcare in the study area is provided by 32 health centres. The secondary level of care within this area, which, among other services, includes the performance of caesarean sections, is available at four rural and one district hospitals. Provincial, central and specialized hospitals, which are located in the two nearest capital cities, constitute the sources of health care at tertiary and quaternary level.

The study’s target population comprised 5 women with fistula confirmed by clinical examination captured during the incidence study from a population of 4441 women who had delivered up to 12 months prior to the start of the incidence study; 9 women with fistula who had delivered before the 12 months but were part of the geographical catchment area of the study (not included in the incidence study) and a matching number of women without fistula (14) who had delivered within the same time period.

### Study design

This qualitative study was carried out using the phenomenological approach, and which, through the use of in-depth interviews, aimed to gain a detailed understanding of individual experiences and the meaning that participants attribute to such experiences with regards to obstetric fistula. The meanings given to these experiences were expressed through their perceptions, beliefs, values and attitudes regarding the phenomenon of interest [19].

### Study procedures

#### Recruitment

**Fig. 1:**
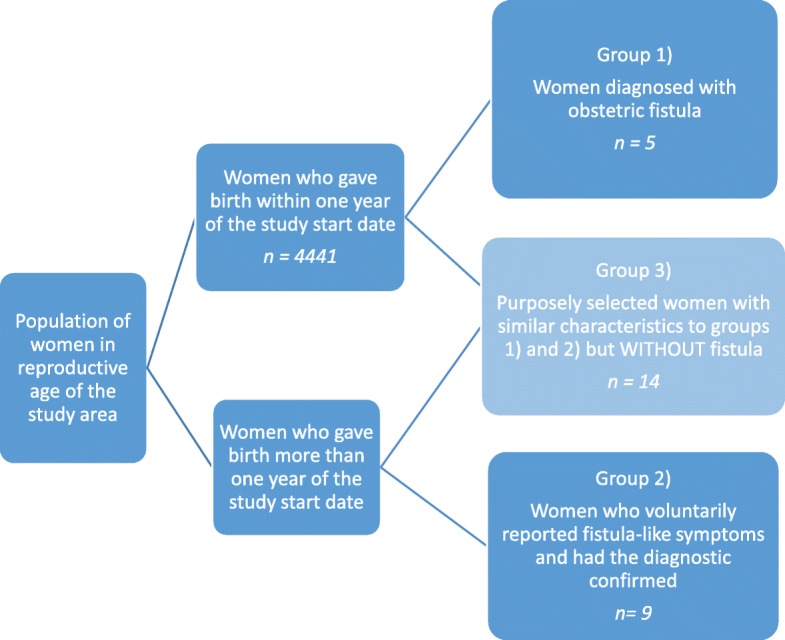
Selection of the three groups of women who took part in the study

Three groups of women were selected to take part in the study: 1) all women with obstetric fistula confirmed by clinical examination from a population of participants in the fistula incidence study [12]; 2) any woman not included in the incidence study but who lived within the study area and had approached the health facility by their own initiative to report symptoms suggestive of fistula and received confirmatory diagnosis; 3) purposively selected women from the same population and with similar characteristics to groups 1 and 2 (matched age and neighbourhood), but without a history of fistula (Fig. 1).

Groups 1) and 2) are referred to as “women with fistula” and group 3) was named “women without fistula”. The latter were included in order to provide a complete description of perceptions and experiences, regardless of the birth outcomes or consequences, in order to capture the uniqueness of the experience of maternal healthcare among women with obstetric fistula by contrasting their experiences with those of women without obstetric fistula.

#### Data collection

Data were collected between August 2016 and March 2017 and consisted of in-depth interviews (IDI). Each interview was conducted by one interviewer and in the most private environment possible. The IDIs followed a piloted topic guide of open-ended questions (Table 1) to allow participants to openly share their experiences with minimal interruption by the interviewer and to allow this process to generate narrative-type answers [20].

**Table 1:** In-depth Interview Guide

How was your experience with your last birth?	
• How old were you when you became pregnant?	
• Did you have any problems during your pregnancy?	
• Did you get an antenatal card?	
Could you tell us about your childbirth experience?	
• What symptoms did you perceive before your labour started?	
• Did you sought any kind of healthcare when the labour pain started?	
• Did you have any complication?	
• What was the result of the pregnancy?	
How did you get to the health facility?	
• How was your experience in the health facility?	
• Have you experienced any delay in receiving care or to attend you?	
Could you tell me about the problem you had after your last childbirth?	
• How did you feel when you realized that you had this complication?	
• How does this problem affect your daily life?	
• Did you seek healthcare when the symptoms started?	
What do you think might have caused this problem?	
• Do you think that your problem could have been avoided? How?	
• How does the community perceive a woman with a fistula?	
How do you see your future after the fistula repair?	
• Do you know how to solve this fistula problem?	
• Do you think you can have children?	
• What do you think that can help you to start a new life after the surgical repair?	
• What are your dreams, expectations for the next 5 years from now?	

Although the guides were written in Portuguese, data collection was conducted primarily in *Changana*, the dominant local language within the study area. The choice of language was determined by the participant’s preference. All but two of the interviews were conducted in the participant’s house. These exceptions occurred to women who belonged to group 2) and were identified and interviewed at the health facility because the interviewer anticipated that it would be challenging to locate their houses for a follow-up encounter. Interviews lasted between 30 and 60 min, and audio recordings of the full interviews were taken. Field notes on the physical and social environment, as well as the actions and reactions of participants and passers-by, were taken to complement the audio information.

The data collection team comprised 2 female interviewers (FM and AM) employed by the Manhiça Health Researcher Centre (CISM). Data collection was overseen by a Mozambican social scientist (HB). This team had worked in previous qualitative studies in the same study area, were familiar with the local context, fluent in Portuguese and *Changana*, and had no prior ties with the participants.

#### Data management and analysis

All audio-recorded interviews were transcribed verbatim. Those conducted in Changana were simultaneously translated to Portuguese during the transcription process. Quality control was ensured by listening to the interviews to confirm accuracy against the written transcripts, followed by immediate feedback to the interviewer and the transcriber. Qualitative data analysis was performed using NVivo version 11.0 (QSR International Pty. Ltd. 2014). A thematic analysis was conducted based on a combination of deductive and inductive coding [21]. Initially, a preliminary coding structure was developed, based on the interview guide questions and pre-determined themes generated from literature and discussions among the project researchers (deductive coding) (see Table 2).

**Table 2:** Initial node structure

Pregnancy experience	
• Ante Natal Care	
• Birth preparedness	
• Complications during pregnancy	
• Nutrition	
• Resources for pregnancy	
Decision making: e.g. decision to seek health care or TBA, or stay to deliver at home, transport searching health care …	
Delivery experience	
• Labour symptoms	
• Complications during labor	
• Experience of pain and fears	
• Type of health facility and health provider	
• Time: to arrive at the facility, of labour duration, or to decide to referral if it was the case	
• Way of delivery	
Health facility assistance	
• Communication with health providers (her perception, experience, emotions), issues of non-response from the health provider, not listening	
• Delays in assistance	
• Issues of disrespect and abuse	
• Referral procedures	
Post-partum experience	
• Consequences	
• Feelings	
• Problems after delivery	
• Symptoms after delivery	
Causes (fistula causes on the woman perspective)	
Relatives attitudes (husband, mother-in-law, mother … other relatives)	
Community attitudes, social insertion (issue of rejection …)	
Support	
Barriers	
Perspectives for the future	
Illnesses or health problems	

Data were coded by two researchers and one outsourced coder, each working independently on their respective NVivo projects, using the same initial coding structures but each with a different set of interviews. Coding was performed by identifying units of text that were meaningful to the study objectives and linking them with the preliminary codes that were representative of those units. At a later stage, new themes identified in the text were linked to additional codes that either branched out from the predetermined codes or constituted completely new ideas (inductive coding).

The researchers convened regularly to maximize coding agreement, discuss emerging themes and definitions, and to interpret and reflect on the findings. Eventually, the three NVivo projects were merged into a single project with a consolidated coding structure (Table 3) and all of the interviews incorporated for the final stage of analysis conducted by one social scientist (HB).

During this stage, similarities and differences within, between and among the different groups of women were compared, and common and divergent patterns of responses were explored in order to capture and ascertain the uniqueness of the experiences of women with fistula.

## Results

Participants’ social-demographic characteristics and their experiences are presented in the format of a descriptive and interpretative narrative, which is subdivided according to overarching themes and sub-themes resulting from single or combinations of selected pre-determined codes. Such results reflect a continuum of events, potentially leading to and resulting from the onset of fistula. The data-driven sub-themes, which mostly reflected participants’ conceptualizations or experiences of fistula and related matters, are highlighted within the narrative text in inverted commas. Direct citations retrieved from the interviews are used to illustrate such conceptualizations.

### Participant’s characteristics

Twenty-eight participants, 14 with and 14 without fistulas, between the ages of 16 and 49 years, were interviewed. Among the group of women with fistula, six were reporting about their first birth experience, 9 had had a caesarean delivery, and 10 had had stillbirths (Table 4).

**Table 3:** Final node structure

Actors	
• Husband or partner	
• Maternal health nurse	
• Medical doctor	
• Midwife or nurse	
• Mother	
• Mother-in-law	
• Nurse Assistant	
• Professional birth attendant	
• Self	
• Técnico de cirurgia	
• Traditional birth attendant	
• Traditional healer	
Barriers	
Decision making	
Eating habits	
Emotions	
Events	
• Ante natal period	
• Birth	
o Delivery	
- Type of delivery	
o Labor	
• Emergency • Post-partum	
Illness or health problems	
• Names or types	
o Fistula	
o Others	
• Perceived causes	
• Perceived health consequences	
• Signs and symptom	
o Pain	
o Urinary incontinence	
Narrative of health care provision	
• Communication with health care provider	
• Practices	
o Instructions	
o Interventions	
o Referral	
• Services	
o Antenatal consultation	
o Maternal waiting home	
o Maternity ward	
o Newborn care	
o Post-partum consultation	
Outcome of pregnancy	
• Live birth	
• Premature birth	
• Stillbirth	
Perspectives for the future Place or provider	
• Community	
• Health facility	
o Central hospital	
o Health center	
o Health post	
o Provincial hospital	
o Rural or district hospital	
• Home	
• On the way to the health facility	
Preparedness	
• Planning	
• Resources	
Social consequences	
• Fulfillment of her role	
• Marital outcome	
Societal attitudes	
• Community attitudes	
• Relatives attitude	
Support	
• Emotional	
• Financial	
Timing Transport

**Table 4:** Participant’s demographic and obstetric information

	Women with fistula *N* = 14	Women without fistula *N* = 14
Age		
16–24	6	6
25–32	5	4
33–38	1	0
39+	2	4
Marital status		
Married	3	14
Single	11	0
Occupation		
Housewife	7	1
Farmer	7	13
Able to read		
No	3	2
Yes	11	12
Able to write		
No	2	2
Yes	12	12
Parity		
1	6	3
2+	8	11
Mode of delivery		
Vaginal	5	13
Caesarean	9	1
Birth		
Live birth	2	14
Stillborn	12	0
Time living with fistula		
1 year or less	5	NA
More than 1 year	9	NA

### Narratives of antenatal and birth care

Study participants had all attended ANC visits (minimum of 3 and maximum of 5). The majority in both groups experienced a normal pregnancy free of disease or complications. Two of the interviewed women with fistula followed the nurse’s advice to stay at the maternity waiting home at the primary health care level during the period around the expected delivery date. Most of the interviewed women reported having planned for a health facility delivery, the majority on their own initiative, and others on the decision of their mothers, mothers-in-law or partners. Planning included mobilizing money to purchase baby clothes, sanitary pads, and *capulanas* (fabrics), as well as to pay a gratuity to the nurses. None mentioned having saved for transport or making other arrangements for referral and counter-referral between the community and the health facility.

All except two (who both gave birth on their way to the health facility assisted by their mothers-in-law) delivered at a health facility.

Most of the women with fistula reported having an obstructed or prolonged birth, which they attributed to a “blockage of the birth canal” or the “baby’s big size”. One woman reported that even after being admitted to the health facility, her relatives sought traditional medicine to ease the expulsion:

*“I first pushed, they [nurses] instructed me to push, so I pushed, but the baby was not coming out, something was blocking the front [the birth canal]. Then the nurses became worried and said “we don’t know what this is.“ Then they called the ambulance. Meanwhile, my mother’s [mother and mother-in-law] had* gone to see a traditional healer and *came back with a remedy that I drank and after that the baby was out …*” *–* Primiparous, Gaza, with fistula for 10 months. Two women with fistula blamed it on “delayed assistance”. Many (10/14) of the women with fistula were readily referred to an upper-level health facility (usually from primary to secondary level). Some (5/14) were further referred to tertiary level, and, for most of these women, the “multiple referral process” plus “delayed assistance at higher levels of care”, took between 1 and 24 h.

*“I was assisted at [name of secondary-level hospital] ... [When] I arrived, they took a long time before performing my surgery … if they had operated on me in the same day that I arrived, maybe my baby would have lived because when I arrived there I was feeling the baby moving … “-* Multiparous, Gaza, with fistula for 2 years*.*

While the majority (13/14) of women without fistula experienced a normal delivery, there was a mixture of vaginal and caesarean section delivery among women with fistula, and those who reported delivering at a secondary or higher level facility had had a ceasarean section.

The women with fistula reported having received more prompt attention and more respect at the primary health facilities compared to that at the higher-level facilities. At the primary level facilities, the women appreciated the prompt arrangement of ambulance for referral, but also the fact that nurses did not shout at them. In contrast, their major complaint regarding the highest level facilities was deficient communication with the health care providers, including being shouted at and not being informed about the procedures they were about to undergo or the reasons for such procedures, giving room for self-interpretations.

*“They just tried to insert a cup [vacuum device] and they could not get the baby out, then they took me to the operating room …*” *-* Primiparous, Gaza, with fistula for 3 months*.*

### Views on pregnancy outcomes

Among women without fistula, all but one had live births. In contrast, the pregnancy outcome for all except two of the women with fistula was stillbirth. Besides the distress due to the fistula itself, the sorrow from experiencing a stillbirth, expressed by them as “no child to bring home”, was recurrent during the interviews. Regarding the perceived causes of stillbirth, a combination of perceived unfortunate obstetric factors and causes related to health services was identified: a woman with fistula reported that the waters broke while she was still at home and the baby died because he ingested her amniotic liquid, referred to as “txupha”; in contrast, other woman with fistula had the sense that the baby was alive upon arrival at the health facility and blamed the death on “delays in performing caesarean section”. *“The problem is that when they referred me, they told me that the baby was still alive …*” – Primiparous, Gaza, with fistula for 3 months.

### Awareness, interpretation and perceived cause of fistula

When asked whether they knew the term “fistula”, most of the women without fistula were not able to explain what it was. However, when the interviewer described the condition, most of them recognized it as “the wetness condition” due to uncontrolled loss of urine, which is referred to in local language as *kuhumessa a mati* (loss of water) and *mavadzi ya ku pfhuta* or *mavabji ya ku pfhutela* (illness of spillage).

When the same question was posed to the group of women with fistula, it was noticed that the term “fistula” was also not known, except for two women who learnt about the name of the condition through the nurse. Most of them stated that they only became fully aware of the problem of fistula through their own experience. In most cases, what triggered this awareness was the abnormal and apparently prolonged post-partum leakage.

*“I did not know what it was, I just felt the leaking and it never stopped.” –* Multiparous, Gaza, with fistula for 34 years.

Alternatively, a few women became aware of some mobility challenges concomitantly with their gradual awareness of the fistula-related leakage. However, they did not seem to associate these symptoms with the presence of a fistula. *“When I looked at myself, I realized that I was no more the same way as I was days ago … I was no more as I was before the symptoms started, I had problems with my leg. I had difficulty to identify the symptoms [of fistula] because I had never experienced it before.” –* Multiparous, Gaza, with fistula for 2 years. Those who experienced fistula and discussed the causes did attribute this condition to the “prolonged and obstructed labour” they had experienced, as well as to “delays in receiving care” and “receiving inappropriate care” at the health facilities. In particular, there was a common perception that the “insertion of a urinary catheter” to assist with urinary retention was harmful, and resulted in perforation.

*“[ …*] *then I stayed there and they inserted a tube into me and when I sat down after removing that tube, I started to have that disease of peeing”* – Multiparous, Gaza, with fistula for 2 years.

Particularly among those who suffered from leg paralysis, there was the perception of it being caused by *xifula* (witchcraft) casted by family members such as the in-laws.

Women who did not experience fistula also attributed fistula to health system factors, including “delays”, “unassisted delivery” and “inappropriate execution of certain procedures”, such as nurses not pulling the baby correctly. Other causes mentioned by them were “damaged” or “spoiled” uterus, “crossing the legs during expulsion” and having had “multiple sexual partners”.

### Experiences of the onset and the course of living with fistula

#### Physical changes and coping mechanisms

The participants with fistula had lived with the condition for a period ranging from 3 months to 9 years, except one woman who lived with this condition for more than 30 years. It took the majority of the participants between a few days and 2 weeks to realize they were leaking urine. Initially, participants viewed it as a normal secretion after delivery, but understood it as a health problem after noticing that the leakage was not ending and was beyond their control. Their main affliction was the discomfort of being wet and the constant need to change clothes.

*“When I arrived home from the hospital I thought the leakage was the discharge that comes out after giving birth but it was not. Then I started to walk always wet, I was always wet and had to change clothes every time”* – Multiparous, Gaza, with fistula for 9 years.

Most of the women wore pads, or pieces of fabric that worked as pads for the leakage to go unnoticed, and reduced liquid intake to minimize the wetting. Despite the perceived gains from the latter measure, they complained that the urine smell became stronger and that its higher concentration exacerbated the burns on the genital area, particularly during summer.

Half of the women with fistula experienced drop-foot, and this seemed to be the trigger for care-seeking at the health facility. Almost invariably they underwent physiotherapy and were prescribed pills, which they took despite not knowing what the pills were. Only one reported having been referred to the quaternary-level hospital for surgery repair, but the surgery did not take place due to the unavailability of the doctor. Those who were taking pills, after realizing that the pills were not effective, interrupted the treatment and did not return to the health facility.

One woman that also had drop-foot explained that it was caused by *xifula* (witchcraft) cast by her sisters-in-law. In her case, the treatment, which was prescribed by traditional healers, consisted of hot foot baths followed by rubbing remedies on fresh razorblade cuts on the skin.

#### Fulfilment of household chores

The combination of urine leakage, pain, discomfort caused by the skin burns, drop-foot and body weakness reduced the women with fistula’s capacity to carry out their basic domestic chores, such as cooking and farming.

Alternatively, women with fistula were less likely to fulfil their responsibilities because they were prohibited from doing so, as expressed by some of the women without fistula, who revealed that women with fistula were expected to be refrained from cooking, grinding, or anything else that would imply hand contact with food or domestic utensils, as there is the perception that women with fistula are “unclean”, and therefore pose a risk of infection to others.

*“This woman with fistula cannot even cook, it is no longer possible. She cannot cook if there are other people in the house. She can only go to Machamba (farm) and fetch water. She cannot grind because she will use her hands … and it is not allowed. “ –* Multiparous, Gaza, without fistula.

#### Social life and support and stigma

Women with fistula reported having to immediately limit their attendance at social gatherings as going to church and participating in funerals. They felt trapped by their situation, not only because of the leakage, but also because of the urine smell. Leakage could be masked by the use of pads, but the pad changing process and what it entailed created additional constraints.

*“I was sad, because I was not free, because I did not go to my neighbours’ house or sleep in relatives’ homes when needed, or otherwise I have to take my pads to secretly and take care of the leakage or I have to leave early to my house to replace the pads.”* – Multiparous, Gaza, with fistula for 34 years.

Those who continued to attend social and religious events had to modify their behaviour as going every time to toilet to change the pads, putting plastic in the bed before sleeping or leaving early. One participant, in tears, said that at the onset of fistula symptoms she had to move out of the house where she lived due to her own sibling’s discomfort with being around her and the mistreatment she received in relation to this attitude.

*“When my sister realised that I had this problem [urinary incontinence] she was no longer close to me, she did not support me at all, she forced me to do domestic work and she would not give me food if I did not work. So I decided to move back to my parents’ house where I am well treated now.”* – Primiparous, Gaza, with fistula for 3 months.

Women without fistula share some views on the issue of social life and support from the standpoint of third parties. For them, although it would not be easy to live with a family member with fistula, they would not reject her and would provide treatment seeking support because she should not be blamed for being in such condition.

*“This person with fistula, being a relative, you have to take care of her because she did not chose, want or enjoy being sick.” –* Multiparous, Gaza, without fistula.

Women without fistula claimed they did not stigmatize women suffering from the condition. Rather, they believed that they should be well treated, but at the same time they stated they should be informed of the things they could or could not do, given their state, and that they understood these restrictions. However, they expressed sympathy with their distress having to bathe every hour and change their clothes, soon after they become wet and smell.

Most women reported not having had sexual intercourse since the onset of the disease. One of them conveyed that her husband considered her to be handicapped because of her condition, considering this a justification to marry a second wife. Women without fistula expressed compassion for women with fistulas and described their “unwell living” and the risk of abandonment by husbands and relatives.

In contrast, more than half of women living with fistula felt the support of their husbands, relatives and community members. This support was demonstrated in terms of family members accompanying them to see conventional or traditional health care providers, or husbands providing financial support to seek care. However, such financial support was not always guaranteed, due to relatives having competing priorities.

### Perspectives for the future

Women without fistula believed that fistula can be cured because “there is nothing that cannot be cured”. Two of them had the notion that the treatment consists of surgery (“to be sewn”).

Almost all of the interviewed women with fistula revealed that they trusted that 1 day in the future they would have the needed medical care (surgery) to repair the damage and defeat the condition. They believed that, because it is a disease that is known by doctors, there must be some form of treatment, despite the impression that it may take some time. They planned to return to their previous social life once the incontinence is resolved. They all hope to fulfil their dreams of continuing with their schooling, work, and enjoyment of motherhood. Many women expressed an interest in having more children, once their obstetric fistula has been properly treated.

*“I always pray to be able to see the future … I have the age to go back to school, I will find a job, besides I did not have the opportunity to study. I know that God is great I will overcome this disease.”* – Primiparous, Maputo, with fistula for 7 years.

Despite this horrific illness and its associated social stigma, most women remained positive, and believed that surgical repair would be made available to women in the future.

*“I will follow by myself [the search for treatment], whether laying down or walking I feel that I will not die like this, I sleep and dream that I will be healed, I will be like other women.”* - Multiparous, Gaza, with fistula for 3 years.

## Discussion

This study, based on narratives generated by in-depth interviews, captured first-hand experiences of women with fistula as well as their counterparts without fistula, enhancing the understanding of the still prevailing obstetric fistula in southern Mozambique. It unravels the contemporary context surrounding a unique generation of women living in an era and a setting within a low-income country where, despite some challenges, reaching a health facility for a safe delivery is possible, and the women themselves, as well as their family members, make every effort to comply with this recommendation (with no distinction as to those with or without fistula), evidenced by their reports on taking appropriate birth preparedness action, staying in maternal waiting homes when needed, and trying to overcome the first-delay barriers. In fact, none of the interviewed women gave birth at home and only two did so on their way to the health facility. Nonetheless, they experienced deficiencies and delays in birth attendance, multiple referrals, and delayed life-saving interventions at the health facilities [12], which are well-documented factors associated with the contraction of obstetric fistula and the worst pregnancy outcomes, such as stillbirths and early neonatal deaths [10, 22]. In this study, the transition from enjoying a healthy pregnancy to a sudden experience of the double burden of obstetric fistula and childlessness is in itself a violence [23]. On top of this suffering, women would have to deal with the incontinence, disability and other complaints linked to a deteriorated health status. Disability was often expressed, not only in terms of physical, but also social limitations, including self, familial and societal denial from most, if not all, of their gender and social roles, be it within intimate, household domestic, and public social domains, all of which stripped them of the essence of being and expressing themselves as women to self, partners, close relatives and society at large [14]. Child loss, which was almost an inevitable aftermath for the vast majority of women with fistula, further increased these women’s sorrow and sense of failure from fulfilling their woman’s role of motherhood [14]. Women’s grief, much present during the interviews, calls for the need for post-repair psychosocial support [13, 24]. Paradoxically, they expressed reliance on some family support, albeit limited just to the treatment seeking process, leaving other aspects of psychological and social support, affection and comfort, uncovered. This isolation and denial from most spheres of their social agency marked their perception that this disease was unique to them, which further refrained them from persuasively searching for a solution. The study captured a polarised situation in regard to the relationships with partners, relatives and community in general, as many women with fistula expressed that they received support from husbands and relatives and that their condition did not necessarily lead to divorce or separation, contrary to findings observed in other studies [4, 13, 24–27]. Nonetheless, this finding should be interpreted with caution, as a remarkable number of women with fistula were registered as unmarried, proving that it is not possible to ascertain a possible connection between the onset of fistula and marital status, while understanding that the definition of marital status in rural settings in Mozambique is challenging. Further research is needed to deconstruct this potential link between marriage dissolution among recently pregnant women and fistula due to its negative consequences on sexual activity.

To our knowledge, this may be one of the first anthropological studies addressing this issue in the context of a new landscape of high facility delivery rates in low-income settings. In this particular study setting, almost 90% of women undergo facility deliveries [12, 28], which is higher than the current national figs [28]. and a substantially higher proportion than what was reported one decade ago in similarly resource-limited countries such as Uganda and Tanzania, where studies addressing obstetric fistula were conducted [4]. It further confirms that such a shift towards facility deliveries per se does not guarantee improved birth outcomes in these settings [29, 30], and looks at this problem from the perspectives of the women themselves, who learnt the hard way about the link between the assistance received and the poor birth outcomes, despite their limited and deficient verbal interaction with health care providers throughout the antenatal, peri-partum and post-partum periods. Our findings are in accordance with previous findings that indicate that women understand that the condition might have been due to prolonged and obstructed labour [22], and that it was aggravated by delayed primary attendance and deficient referral and post-referral care [31]. Further, some women did view their fistula as being iatrogenic, as previously reported [4].

This study provided a dissimilar finding in relation to what was previously reported, where fistula cases were almost consistently those who had experienced severe first or second delays [3–5]. In the present study, most had managed to beat the first and second delays, but all of the women with fistula reported the occurrence of the third delay in receiving care to prevent the reported adverse outcomes of pregnancy [12], and all experienced delays in appropriate care in response to the perceived symptoms of fistula (the fourth delay) up to the moment of the interview, as previously reported [4, 5]. While prompt and satisfactory attention was reported at a primary level of care, women reported experiencing challenges suggestive of negligence, and some verbal abuse at referral level, but not physical abuse, as reported in other settings [4, 32] . However, this is still of concern and can be considered as concordant with other studies, if negligence is considered as a dimension of obstetric violence [33]. In contrast, women without fistula did not report such negative experiences with the health services, suggesting these to be unique experiences linked to women undergoing complications, leading to fistulas.

Unexpectedly, and despite their disappointing experience with the assistance received thus far, the women with fistulas revealed having some hope for an eventual cure, which would then contribute to the achievement of future personal plans. This is encouraging, given that this implies their openness to continue seeking care at the health services which earlier failed to assist them. This regaining of trust must be accompanied by an adequate response from the health services.

The interviews with women with similar characteristics but without fistula enabled a deconstruction of what separated the experiences uniquely bound to women with fistula from what women in general might go through during the antenatal, peri-partum and post-partum periods in this particular setting. Those without first-hand experience of fistula had little insight into the problem and expressed a puzzling position of both blaming the poor quality of health care provision and the attitudes of the women themselves, while having sympathy for those suffering from the condition. Although not opposed to the social, often stigmatizing, restrictions that the women with fistula are exposed to, the interviewed women without fistula expressed empathy and a sense of moral obligation to support women affected by fistula. This confusion might be due to the rare occurrence and even less so discussed incidence of 1 fistula case per 1000 births in this setting [12], combined with the negative influence of societal prejudices on the attempts to understand the unknown. As the views of women without fistula partly provide insights into the society in which the women with fistula live, it becomes apparent that increased consciousness at a societal level about the meaning and implications of living with fistula, as well as the needs of women with this condition, is needed. This calls for further health promotion efforts, moving from just focusing on appropriate care-seeking practices during pregnancy, delivery and post-partum, to also include community-based demystification of the current misconceptions about the causes of fistula. From the point of view of the health services this study reveals the need to supporting facility-based prevention, early detection and treatment provision, as well as the consideration of modification in the referral guidelines to allow for primary to tertiary or quaternary level referrals in extreme cases in order to address the third and fourth delays.

## Methodological considerations

The population-based case-finding approach, as opposed to the earlier documented health-facility-based recruitment methods [20, 26], as well as the possibility of conducting the interviews outside of the health facility and separately from any fistula-related medical assistance that the women might have been going through, was a strength of the study.

We faced several limitations. First, the depth of some discussions was limited due to the emotions evoked during the questioning. The interviewer had to modify the conversation to prevent further emotional distress, which narrows the potential that phenomenological studies have to obtain in-depth insights into participants’ reported experiences and the meaning of such experiences. Secondly, participants were interviewed after being informed about a possible surgical repair, which may have affected their perspectives on fistula in general and about their future in particular. Social desirability may have affected what women expressed, as the interviewers were not health professionals but were linked to the team who offered the possibility of care for the fistula condition.

Interviewers were well trained, equipped with previous qualitative research experience, familiar with the community, and fluent in the local language. Relationships with the communities were established prior to data collection by approaching the administrative post chiefs, traditional leaders, and the neighbourhood secretary for prior permission. Credibility was promoted by engaging in regular discussions during the data collection and interpretation of the findings within the research team, each being drawn from different perspectives and expertise. The study design, including the population case-finding recruitment method, and early case-finding in the first year postpartum, was also considered a strength of the study by enabling the early capturing of the onset of obstetric fistula. By interviewing women without fistulas, we also began to gain a sense of community perceptions and attitudes. The results refer to the specific context of the Mozambican healthcare system, but we would expect similar experiences in other rural African countries with a similar socio-cultural and health system context. Efforts were made to provide a detailed description of the context in relation to the setting, the sampling of informants, and the interpretation of the results to assess the transferability of the results to other low-income settings. However, due to the nature of the methodology, any possibility of transferability must be determined by the reader. One significant strength was that we took advantage of this being a rare event in the setting in order to interview all, or virtually all, cases, which produced a theoretical generalization of the situation of women with fistula in this area (two entire provinces).

## Conclusion

Although women do seek antenatal and peri-partum care in health facilities, deficiencies and delays in birth assistance, referral and life-saving interventions were commonly reported by women with fistula. Furthermore, weaknesses in quality of care, not only in relation to prevention, but also the resolution of the damage, were evident. Quality improvement of birth care is necessary, both at primary and referral level. There is a need to increase awareness and develop guidelines for prevention, early detection and management of obstetric fistula, including early postpartum treatment, availability of fistula repair for complex cases, and rehabilitation, coupled with the promotion of community awareness of the problem.

## Data Availability

The datasets used and analysed during this study will be stored at the CISM repository and are available on request by contacting the corresponding author, provided compliance with the CISM data sharing and property policy is upheld.

## Abbreviations

ANC: Ante-Natal Care
CISM: Manhiça Health Research Centre
IDI: In-Depth Interview
LMIC: Low- and Middle-Income Countries
